# Low-Temperature Induced Enhancement of Photoelectric Performance in Semiconducting Nanomaterials

**DOI:** 10.3390/nano11051131

**Published:** 2021-04-27

**Authors:** Liyun Wu, Yun Ji, Bangsen Ouyang, Zhengke Li, Ya Yang

**Affiliations:** 1 School of Material Science and Engineering, Sun Yat-Sen University, Guangzhou 510275, China; Wuly37@mail2.sysu.edu.cn; 2 Center for Excellence in Nanoscience, Beijing Key Laboratory of Micro-Nano Energy and Sensor, Beijing Institute of Nanoenergy and Nanosystems, Chinese Academy of Sciences, Beijing 101400, China; jiyun@binn.cas.cn (Y.J.); ouyangbangsen@binn.cas.cn (B.O.); 3 School of Nanoscience and Technology, University of Chinese Academy of Sciences, Beijing 100049, China; 4 Center on Nanoenergy Research, School of Physical Science and Technology, Guangxi University, Nanning 530004, China

**Keywords:** temperature dependence, photoelectric, photodetector, solar cell

## Abstract

The development of light-electricity conversion in nanomaterials has drawn intensive attention to the topic of achieving high efficiency and environmentally adaptive photoelectric technologies. Besides traditional improving methods, we noted that low-temperature cooling possesses advantages in applicability, stability and nondamaging characteristics. Because of the temperature-related physical properties of nanoscale materials, the working mechanism of cooling originates from intrinsic characteristics, such as crystal structure, carrier motion and carrier or trap density. Here, emerging advances in cooling-enhanced photoelectric performance are reviewed, including aspects of materials, performance and mechanisms. Finally, potential applications and existing issues are also summarized. These investigations on low-temperature cooling unveil it as an innovative strategy to further realize improvement to photoelectric conversion without damaging intrinsic components and foresee high-performance applications in extreme conditions.

## 1. Introduction

To meet the emergent requirement of the development of sustainable and clean energy, solar energy, recognized as the best potential alternative to fossil-fuel power, has been intensively investigated regarding its effective harvesting, conversion and storage in past decades [1,2,3,4,5,6,7,8,9]. One of the major developments lies in the mature utilization of nanoscale materials, such as two-dimensional materials [10,11] and quantum dots [12]. However, the advent of the photoelectric device also raises concerns regarding adaptivity to diversified environments, such as outer space or other extreme environments. Differing from the widely researched performance at normal temperature condition, the investigations on extreme temperature conditions, especially low temperature, are insufficient for completing the underlying physical mechanism and applying it to practical use. There are a handful of reports showing decreased performance at low temperature. These weakening phenomena caused by lowering temperature are ascribed to many causes including frozen carrier diffusion [13], reduced absorption [14], interfacial ion accumulation [15], phase transition [16] and radiative [17] and nonradiative [18] recombination. However, there are also many reports that low-temperature operation is not a restriction but an enhancement to overall photoelectric performance, ranging from low-dimension materials to nanocrystals. The effectiveness of the cooling method on these devices not only provides a strategy for constructing photoelectric devices under extreme conditions but also reveals the potential of cooling as an innovative and efficacious method for improving optoelectronic performance. It is worth noting that small drops in temperature will also cause visible improvement in photoelectric performance [19,20,21]. No matter the need, in applications or in theoretical research, relevant reports on the cooling method is in urgent need of systematic summary and guidance.

**Figure 1:**
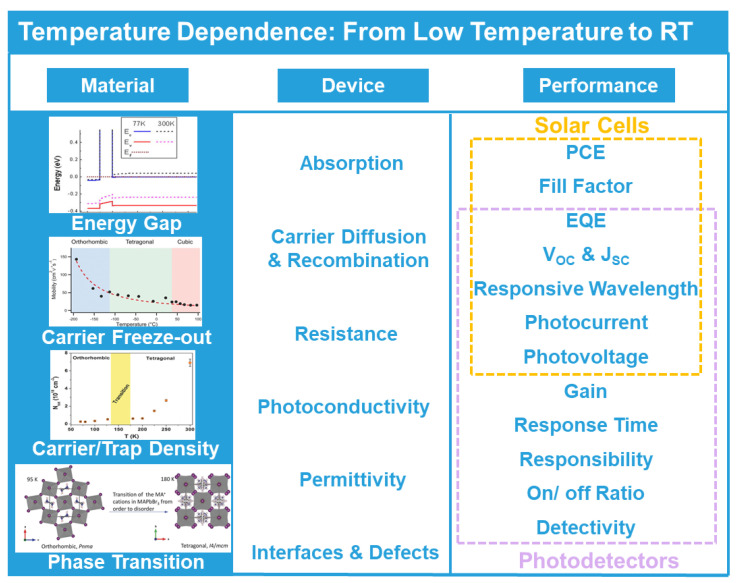
Temperature-dependent effect on photoelectric devices including materials, devices and performances. All images reproduced with permission: “Bandgap” (adapted from [29], with permission from the American Institute of Physics, 2014). “Carrier freeze-out” (adapted from [30], with permission from Wiley-VCH, 2015). “Carrier/Trap Density” (adapted from [31], with permission from Wiley-VCH, 2017). “Phase Transition” (adapted from [32], with permission from Wiley-VCH, 2018).

To realize improvements through low-temperature cooling, the underlying function mechanism deserves investigation. Similar to coupling effects or surface modification [22,23,24,25,26], the cooling method functions without changing composition or inherent structures. Moreover, compared with these traditional methods, cooling’s enhanced effects have unique advantages in applicability, stability and nondestructive characteristics. In view of the existing theories, the general temperature-dependent methods can be divided into three aspects: materials, devices and performances, shown in Figure 1. Temperature variation first directly functions on materials, inducing fluctuations in energy level [27,28,29], carrier motions [30], density of traps/carriers [31] and crystal structure transition [32]. For cooling-enhanced materials, regulation in energy levels causes changes in absorption [28], barrier height [33] and so on [34,35], then influences photon absorption and charge transportation. Some intrinsic characteristics of carriers are reported to show huge enhancement from cooling, including mobility [36], diffusion length [37] and corresponding densities [38]. For specific materials, structure transition [39] has been observed in a low-temperature range, providing more potential for cooling regulation. When the above phenomena integrated with devices, the cooling-enhanced properties in devices can be classified into absorption [40], carrier diffusion and recombination [41,42], resistance [43], photoconductivity [44], permittivity [45], interfaces and defects [46] and can improve the overall performance of both photodetectors and solar cells. The full mechanism is based on temperature-dependent optical and electrical properties, thus the emergence of the utilization of the cooling method, which possesses many advantages over conventional improvement methods and also possesses benefits to reliable photoelectric application in harsh environments.

Here, we summarize the cooling-enhanced effect on the photoelectric process in various nanoscale semiconductors from near room temperature (300 K) to liquid nitrogen temperature (77 K). First, low-temperature-enhanced photoelectric parameters, materials and their corresponding devices’ structures are classified for further analysis with the underlying photoelectric mechanism. On this basis, the cooling-enhanced photoelectric performance and corresponding physical mechanism are demonstrated in different photoelectric processes. In this section, we also symmetrically discuss the related characteristics of cryogenic-enhanced optoelectronic devices from carrier movement to device optimization. Finally, the future opportunities and potential application of cooling-enhancing effects in optoelectronics are discussed for further scientific research and practical utilization.

## 2. Materials with Cooling-Enhanced Effects

**Figure 2:**
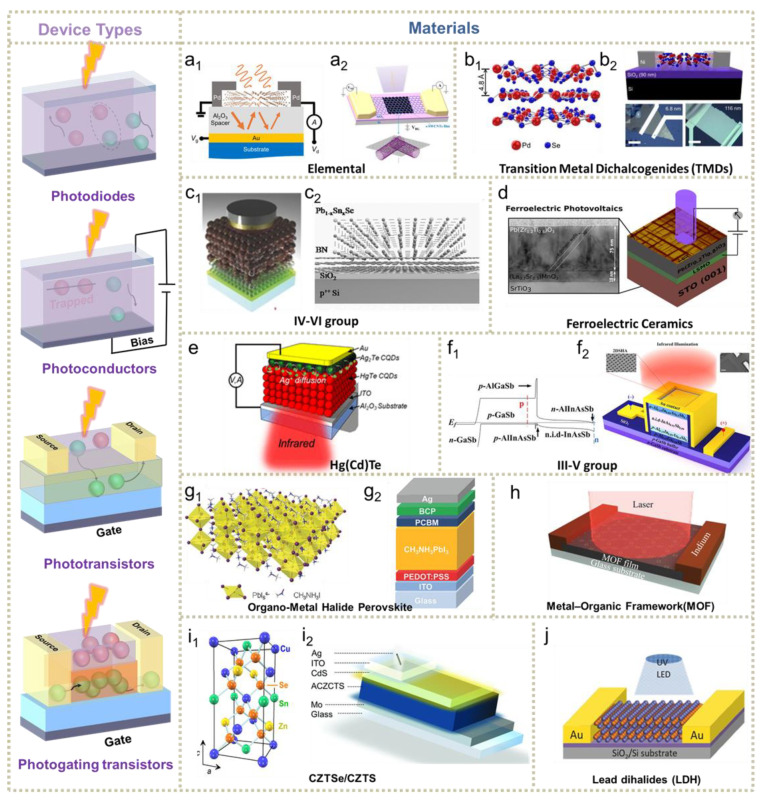
Fundamental devices configuration and materials categories with cooling-enhanced photoelectric performance. (**a**) Elemental semiconductors. (**a_1_**) Tellurium nanoflakes (adapted from [47], with permission from the American Chemical Society, 2018). (**a_2_**) Graphene (adapted from [48], with permission from the American Institute of Physics, 2018). (**b**) Transition metal dichalcogenide (adapted from [49], with permission from the American Institute of Physics, 2019). (**c_1_**) PbS (adapted from [50], with permission from Wiley-VCH, 2014). (**c_2_**) Pb_1−x_Sn_x_Se (adapted from [51], with permission from Wiley-VCH, 2015). (**d**) Ferroelectric ceramics (adapted from [52], with permission from the American Chemical Society, 2020). (**e**) Hg(Cd)Te (adapted from [53], with permission from the American Chemical Society, 2018). (**f**) III-V group. (**f_1_**) Band alignment of p-i-n structure. (**f_2_**) p-i-n structure (both adapted from [54], with permission from the American Institute of Physics, 2018). (**g**) Organo-metal halide perovskite. (**g_1_**) Crystal structure (adapted from [55], with permission from Wiley-VCH, 2016). (**g_2_**) Planar perovskite solar cells (adapted from [31], with permission from Wiley-VCH, 2017). (**h**) Metal-organic framework, reproduced with permission [20], from Wiley-VCH, 2020). (**i**) CZTSe/CZTS solar cells. (**i_1_**) Crystal structure (adapted from [56], with permission from Elsevier, 2017). (**i_2_**) CZTS planar solar cells (adapted from [57], with permission from Wiley-VCH, 2018). (**j**) Lead dihalides (adapted from [58], with permission from Wiley-VCH, 2017).

The influence of temperature on the photoelectric process is closely related to nanomaterials and their structures. The working range of the cooling method includes nanomaterials from elemental semiconductors to organic-inorganic complexes. The reported works on cryogenic-enhanced optoelectrical performance are based on various photoelectric structures, summarized as photovoltaic photodiodes, photoconductors, phototransistors and photogating transistors, as depicted on the right side of Figure 2.

In photovoltaic photodiodes, effective electron-hole pairs are activated by absorbed phonons, and then separated by built-in electric fields to transport to specific electrodes for collection. In this case, Ohmic contact, Schottky contact, the p-n junction or more complex p-i-n junction are required driving forces. Solar cells [13,59,60], III-V group infrared photodetectors [61,62,63,64,65,66], as well as most ferroelectric ceramic [67,68,69,70,71]-based devices, have evolved from photovoltaic photodiodes. Another popular photoelectric device is the photoconductor, which responds to illumination through photoconductance changes. By sacrificing the self-powered characteristic, photoconductors have superiorities in gain (G) [72,73] by applied bias voltage due to bias-enhanced carriers’ lifetime. In cryogenic enhancement tests, the photoconductive type is widely employed in two-dimensional materials [74] and quantum dots (QDs) [33,75,76,77]. The main problem faced by photoconductive devices is comparatively higher dark current. The device consists of terminals played by source, drain and gate, and photoactive channel to lower dark current and increase photoresponse in phototransistor structure. When integrated with high mobility materials, two-dimensional materials [20,78,79] prefer to adopt phototransistor structure. As a special example in phototransistor devices, photogating phototransistors stem from the photogating effect, which modulates devices’ conductance by phonon-induced traps. Several graphene-based structures [48,80] have been explored as cooling enhanced their photogating performance. Here, we focus on photoelectric devices with application in solar cells and photodetectors, especially infrared (IR) detectors consisting of near-infrared (NIR, 0.7–1 μm), short-wavelength infrared (SWIR, 1–3 μm), mid-wavelength infrared (MWIR, 3–5 μm) and long-wavelength infrared (LWIR, 8–14 μm) photodetectors. On the basis of devices’ structures, numerous materials, simply classified in the right part of Figure 2, varying from elemental to inorganic-organic hybrid semiconductors, have been researched for low-temperature photoelectric performance. As shown in Figure 2a, the elemental materials are represented by graphene [33,81,82,83], black phosphorus [84,85], silicon [86], tellurium [47] and boron [87]. Since these 2D materials prefer the photoconductive mechanism, the utilization of the cooling method mainly influenced their photoconductive characteristics for devices’ performance. Two-dimensional transition-metal dichalcogenides (TMDs) [88,89]-based photodetectors have also been intensely explored in visible and near-IR spectrum. They are competitive in high stability, high photoresponsivity and quick response. Zhang et al. studied PdSe_2_-based photodetectors’ performance from 77 K to 300 K and found higher effective electron mobilities up to 520 cm^2^ V^−1^s^−1^ at 77 K, further improving photoresponse [49]. In addition to elemental quantum dots, IV-VI group materials, represented by PbS [50,90,91], PbSe [19,51] and PbTe [92], have been tested for low-temperature photodetection on solar cells (Figure 2c). Speirs et al. found PbS quantum dots solar cells’ FF and V_OC_ increased when cooling to 230 K without sacrificing current, resulting from temperature-dependent reverse saturation current [93]. Chusnutdinow et al. explored PbTe-based photodetectors that performed better as 2.7 × 10^10^ Jones under low temperature for raised carrier intensity and mobility [92]. Weng et al. prepared a CdS/PbSe photovoltaic photodetector and then discovered that the cooling-enhanced cutoff wavelength expanded to near 7.0 μm due to the band energy component being changed by the temperature [19]. As depicted in Figure 2d, the unique photovoltaic effect in ferroelectric materials like BiFeO_3_ [70,94,95,96,97,98] and Pb(Zr,Ti)O_3_ [52,99] inspired research on temperature-influenced bulk photovoltaic performance. The research of III-Vs and HgCdTe-based photodetectors significantly pushed forward the development of infrared sensing systems. HgCdTe-type IR photodetectors were predicted to be more qualified than both MWIR and LWIR for their long S–R lifetime near 4000 us [100], compared with IV-IVs. Zhu et al. investigated the temperature-dependent relationship between low frequency noise and dark current in SWIR, MWIR and LWIR [101]. Due to the competition between diffusion and generation-recombination (G-R) current, declining temperature can effectively reduce dark current. Another newly emerging Hg(Cd)Te-type infrared photodetector is HgTe QDs for the MWIR range. Ackerman et al. constructed HgTe CQDs that performed 0.56 A W^−1^ at 150 K for low-temperature enhanced diffusion [102]. For the III-Vs group, as illustrated in Figure 2f, the p-i-n structure is commonly adopted to reduce noise current with higher barrier height. Apart from designing these complex structures, cryogenic cooling methods have been widely proven to be effective in weakening dark current in III-Vs photodetectors, which will be further discussed in Section 3.2.2. It is necessary to emphasize that the intensive investigation of organic-metal halide perovskite solar cells (Figure 2g), where the organic part refers to methylammonium (MA) cations and formamidinium (FA) cations, gives in-depth insight to the mechanisms of the temperature-dependent photovoltaic effect from material to device, deeply discussed in Section 3.3. As a tailorable electric material, MOFs with semiconducting features are capable of functioning as active elements in the photoelectric process. Recently, Arora et al. reported a novel Fe_3_(THT)_2_(NH_4_)_3_ MOF-based photodetector presenting an improved photoresponse with declining temperature [20]. Authors attributed these phenomena to suppressed thermally activated carriers under 77 K. CZTS or CZTSe is another promising thin-film material for solar cells owing to its tunable energy gap and low-effective charge masses [56]. Hadke et al. characterized the temperature-dependent behavior V_OC_ of several CZTS devices and discovered that all of them increase with decreasing temperature [57], resembling other photovoltaic cells [86,93,103,104]. Similar to TMDs, LDHs are newly developed photoconductive 2D materials with superiority in low dark current and ultrahigh photoresponse. As a representative LDH material, PbF_2−x_I_x_ has been reported with cooling-enhanced photodetection performance caused by less phonon scattering under low temperature [58]. It is worth noting that low-temperature enhanced effects have been reported on the above popular optoelectronic materials, which is especially beneficial to low-dimension semiconductors.

## 3. Low-Temperature Enhancement in Photoelectric Performance

The interplay between temperature and the photoelectric process varies along with the specific design of devices. We divided low-temperature-enhanced performance into three parts to analyze the specific function of cooling effects: common physical parameters represented by absorption, photodetector performance, and solar-cell performances. Moreover, the related formulas of temperature-dependent photoelectric parameters are listed in Table 1.

### 3.1. Absorption

**Figure 3:**
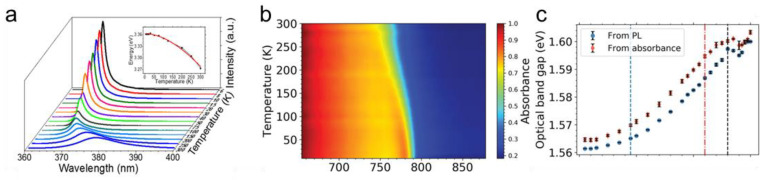
Cooling-enhanced phenomenon of absorption. (**a**) Temperature-dependent absorption of p-Si/n-ZnO photodetector. Inset: bandgap at various temperatures (adopted from [113], with permission from the American Chemical Society, 2017). (**b**) Temperature-dependent absorption of perovskite solar cells (adapted from [39], with permission from Wiley-VCH, 2019). (**c**) Temperature-dependent optical bandgap of perovskite solar cells (adapted from [39], with permission from Wiley-VCH, 2019).

Despite photoelectric investigations over past decades, optical properties like absorption are bottlenecks restricting semiconductor candidates in both photodetectors and solar cells. Unlike traditional tuning methods by doping or sensitizing [110,111,112], coupling effects and surface modification are reported effective without changing nanostructures. However, they are hindered by applicable materials, performance stability and potential damage. The cooling method can compensate for these. In 1967, Varshni proposed a theoretical equation to elucidate the relationship between bandgap and operating temperature based on the theory of electron-phonon interactions and lattice expansion [105]. The full formula is listed in Table 1 where E_g_(0) is the bandgap of the semiconductor at 0 K, while α and β are empirical constants. According to the Varshni equation, the cutoff wavelength of devices based on single semiconductors tends to monotonous blueshift. As shown in Figure 3a, a significant blueshift of absorption and a widened bandgap is observed in the p-Si/n-ZnO heterojunction when starting temperature declines [113]. However, as shown in Figure 3a,b, for both photoluminescence (PL) spectra and absorption spectra, the (FAPbI_3_)_0.85_(MAPbBr_3_)_0.15_ perovskite thin films show a red-shift trend [39]. Thus, a declined optical bandgap at low temperature is calculated. Similar positive relationships between bandgap and lowering temperature have also been reported in other hybrid perovskite [41,114,115,116] materials, since the overlapping orbitals of halogens [34] caused reductions in the valence band. Another cause for the broadening spectra of hybrid perovskites is phase transition [117,118,119] during cooling. Zheng et al. reported the unusual redshift in absorption attributed to phase transition between orthorhombic and tetragonal structures [119]. Moreover, the phenomenon of widening absorption using cryogenic experiments also exists in other materials and structures. Szendrei et al. witnessed a narrowing bandgap along with cooling in PbS nanocrystals [120], consistent with the previously observed size-dependent dE_g_/dT effect [121] in PbS quantum dots. Black phosphorus [28,122,123] is also observed to have an abnormal temperature-dependent effect. A comprehensive investigation on n-CdS/p-PbSe photodetectors found that the cutoff wavelength increases with declining temperature [19]. Authors reasoned that temperature caused the energy band gap of PbSe and CdS mismatching and shifted the band alignment from type II to type I. Likewise, the tuneable band alignment of the p-n junction under temperature intervention has been studied in other structures, such as GaTe-MoS_2_ [124] and SnS_2_/p-Si [125].

According to the Varshni equation (bandgap equation in Table 1), the absorption coefficient exhibits similar temperature-dependent properties. As illustrated in Figure 3c, even the cutoff wavelength narrowed at lower temperature. The InAs/InAsSb pBn photodetector at cryogenic temperature yielded a higher quantum efficiency at the specific wavelength [126]. Authors proposed a relationship between the absorption coefficient and the temperature-influenced bandgap as:

(1) $$\alpha \left(\gamma \right)\propto {\left[E_{\lambda }-E_{g}\left(T\right)\right]}^{1/2}$$

From this equation, bandgap variation crucially influences the absorption coefficient during cooling. Nevertheless, carriers’ diffusion and recombination occupy a crucial place in the absorption coefficient at various temperatures. In the inset picture of Figure 3c, lower quantum efficiency above 220 K has also resulted from the increased Auger recombination rate and impeded minority diffusion at the higher temperature.

### 3.2. Performance of Photodetectors

Originating from research on light-to-electric conversion, photodetectors are an appealing field in optoelectronics, owing to their applications in optical communication, healthy imaging, security monitoring and fire/gas sensing. Similarly to radiation intensity and built-in electric fields, temperature-dependence also exists in the photodetection process, which deeply influences the key processes in light-electric conversion.

In addition to the response spectra discussed above, detectivity and responsivity, as well as quantum efficiency, are the main key figures of merit for photodetectors. Responsivity is proportional to carriers’ mobility and the density of photogenerated carriers, shown as:

(2) $$R=\frac{{q\eta \tau }_{l}}{{\mathrm{hv}\tau }_{t}}=\frac{{q\eta \tau }_{1}\mu V}{{\mathrm{hvL}}^{2}}$$

In this formula, η represents quantum efficiency, τ_1_ means the lifetime of photogenerated charges, μ is mobility, V and L are applied bias voltage and channel length, respectively [58]. Likewise, other figures of merit also heavily relied on temperature for intrinsic parameter variation. Nevertheless, considering different photodetection mechanisms, the cooling-enhanced effect functions with different pathways, and we will discuss them in categories.

#### 3.2.1. Photoconductive Photodetectors

**Figure 4:**
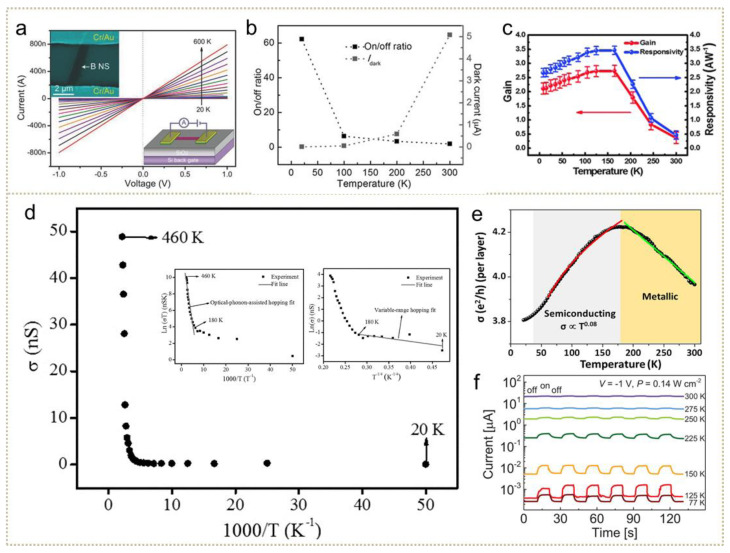
Cooling-enhanced phenomena on photoconductive photodetectors. (**a**) Temperature-dependent *J-V* characteristics of ultrathin boron nanosheets (adapted from [87], with permission from Wiley-VCH, 2015). (**b**) Temperature-dependent on/off ratio and dark current in Ta_2_NiSe_5_ nanoflakes (adapted from [129], with permission from Wiley-VCH, 2016). (**c**) Temperature-dependent gain and responsivity in Sb_2_Te_3_ photodetectors (adapted from [128], with permission from the Royal Society of Chemistry, 2015). (**d**) Schematic of photoconductivity variation with temperature (adapted from [130], with permission from Wiley-VCH, 2017). (**e**) Photoconductivity of graphene (adapted from [74], with permission from the American Chemical Society, 2020). (**f**) Current variation under cycled light at different temperatures (adapted from [20], with permission from Wiley-VCH, 2017).

For the photoconductive mode, photoresponse relies on the process of light absorption in semiconductors raising excess free charges then causing increments in conductivity to realize the sensing process. An additional key figure of merit of photoconductive photodetectors is gain (G) [3], defined as:

(3) $$G=\frac{I_{\mathrm{ph}}}{q}\left(\phi_{\mathrm{in}}\mathrm{QE}\right)=R\frac{\mathrm{hc}}{\eta q\lambda }$$

where R means responsivity, η, q, λ, h and c represent quantum efficiency, the absolute value of electron, illuminated wavelength, Planck’s constant and the velocity of light, respectively. G represents the ratio of the photogenerated carriers collected by the electrode to the photons absorbed by the semiconductor. Likewise, G showed enhanced performance with cooling [127], directly related to carriers’ lifetime. The temperature effects on photoconductive photodetectors supposedly originated from photoconductivity, consisting of charges’ separation, recombination and diffusion processes. Then, temperature functioned on the overall performance, like on-off ratio, responsivity, G and more. Here, we take several typical parameters of photoconductive photodetectors as an example. In Figure 4a, a typical *J_dark_-V* curve of a boron-nanosheet-based photodetector is measured at temperatures from 20 to 600 K [87]. As extracted from the slope of the *J-V* curves, it can be concluded that resistance decreases when temperatures rise. Another report on Ta_2_NiSe_5_ nanoflakes was performed with an enhanced on/off ratio and suppressed dark current, depicted in Figure 4b. The on/off ratio was significantly enhanced from ~0.1 at 300 K to ~60 at 25 K, realizing reliable signal identification for 808 nm light. Moreover, the resistance increased to 35 MΩ at 25 K, meaning dark current was hampered heavily at low temperature. This phenomenon indicates the immense potential of low-temperature operation in improving the on-off ratio and strengthening light-generated current as well as further enhancing responsivity and gain considerably. However, even with cooling the hampered photocurrent, there are photoconductors performing with higher gain and responsivity at low temperature, rather than room temperature, shown in Figure 4c. Zheng et al. synthesized the topological insulator Sb_2_Te_3_ photodetector. The enhanced gain and responsivity are acquired by cooling. Simultaneously reduced photocurrent is resulted from competences of bulk states and surface states at different temperatures [128].

To comprehend the underlying mechanisms of temperature variation and photoconductive performance, temperature-dependent conductivity (σ), which is dominated by the interaction between phonons and carriers or polarons, has been widely investigated. As show in Figure 4d as an example and guided by Mott’s model [108], the plot of conductivity inverse temperature can be divided into two fitted near-linear curves representing nearest neighbor hopping (M-NNH) and the variable range hopping mechanism (M-VRH), respectively. For the GeSe_2_ photoconductor [130], the nearest neighbor hopping mechanism dominates conduction variation from 180 to 460 K, and variable range hopping is fitted to conduction changes from 20 to 180 K. In the nearest neighbor hopping region, the hopping of small polarons is assisted by optical phonons. The activation energy for the hopping conduction mechanism has been proposed by Austin and Mott [131,132], as follows:

(4) $$W=W_{D}+\frac{1}{2}W_{H},\;T>\frac{1}{2}\theta_{D}$$

(5) $$W≅W_{D},T<\frac{1}{4}\theta_{D}\;\;$$

where θ_D_ is Debye temperature, W_H_ and W_D_ represent hopping energy and disorder energy, respectively. As the temperature drops further, the optical phonons’ energy becomes inadequate to maintain high conduction. Thus, the variable hopping mechanism, assisted by single acoustic phonons, is suitable for low temperature. There are a handful of experiments that evidenced this conduction-temperature model as accounting for temperature-dependent sensing performance, such as HgTe QDs [133], PbS/polystyrene nanocomposite [77], boron sheet [87], InI [134] and monolayer graphene [79]. What deserves notification are conduction changes raised by phase transition, which needs to be analyzed based on specific situations. In Figure 4e, Mogera et al. investigated the temperature-dependent conductivity of twisted multilayer graphene and observed peak conductivity appearing at ~180 K, indicating semiconducting to metallic transition [74].

Moreover, there are still challenges for photoconductive light sensors to overcome the huge dark current at room temperature. This issue can be effectively solved with the proper cooling method, such as, for example, Fe_3_(THT)_2_(NH_4_)_3_ MOF-based photodetectors (Figure 4f). The photoswitching response of their MOF devices at various temperatures possessed different on/off ratios, reaching a maximum at 125 K [129]. The response to light with long wavelengths and weak power intensity can appear and increase at cooling temperatures. Huang et al. reported the phenomenon that black-phosphorus-based photodetectors show a high responsivity of 7 × 10^6^ A W^−1^ at 900 nm laser with an intensity of 5 mW cm^−2^ [78], exceeding the detection limit at room temperature. Another research on Ge QDs photodetectors observes an extraordinary and obvious photoresponse to near-IR light with only 10 nW of light intensity [135]. The enhanced weak light response is likely due to the integrating function of suppressed noise current and enhanced absorption.

In addition to photoconductive devices’ performance, low temperature enhances carriers’ inherent movement, evaluated by lifetime, mobility and concentrations simultaneously. There are also investigations covering these specific carriers’ characteristic changes under variations in temperature. According to the definition of gain, carrier lifetime can be reflected by the calculated gain. A relationship between effective mobility and the temperature extracted from Te-based FET is fitted with μ_Eff_∝T^−1.03^ [47], corresponding with the phonon-scattering model [136]. However, the positive influence of temperature on mobility is not infinite. Radisavljevic et al. systematically measured the charge transport properties of monolayer MoS_2_ devices. At temperatures above 200 K, mobility follows μ~T^−1.4^, indicating a typical phonon-scattering limited behavior. With further decreases in temperature, mobility shows pronounced dropping due to charged impurities scattering. In this structure, the impurity scattering has been screened by depositing the top-gate dielectric.

#### 3.2.2. Photovoltaic Photodetectors

**Figure 5:**
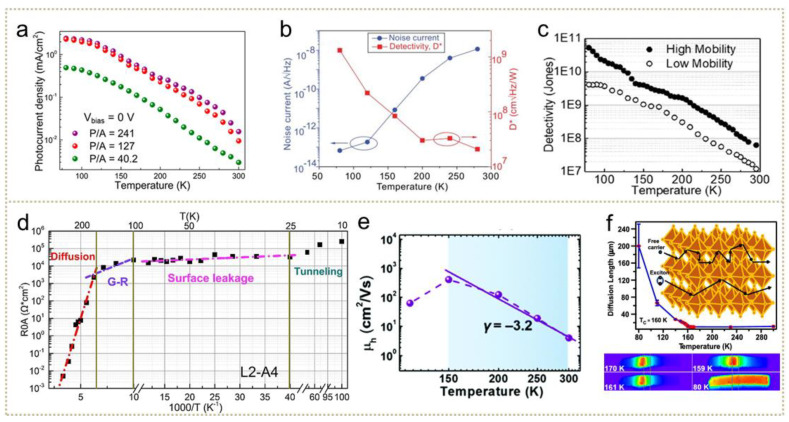
Cooling-enhanced phenomena on photovoltaic photodetectors. (**a**) The temperature-dependent photocurrent density of InSb photodetectors (adapted from [137], with permission from the American Chemical Society, 2018). (**b**) The low-temperature-enhanced detectivity and noise current of II-VI quantum well infrared photodetectors (adapted from [35], with permission from the American Institute of Physics, 2013). (**c**) The temperature-dependent detectivity of HgTe CDs photodetectors (adapted from [133], with permission from the American Chemical Society, 2019). (**d**) The temperature-dependent R_0_A of InAs/GaSb photodetectors (adapted from [139], with permission from Elsevier, 2017). (**e**) Temperature-dependent *I-V* characteristics and the corresponding mobility of perovskite photodetectors (adapted from [140], with permission from the Royal Society of Chemistry, 2018). (**f**) Temperature-dependent carriers’ diffusion length of perovskite photodetectors (adapted from [37], with permission from the American Chemical Society, 2020).

Different from the photoconductive type, the photovoltaic-type photodetector is based on the generation, diffusion and collection of photogenerated carriers, and performance is mainly determined by photogenerated charge pairs. A crucial factor affecting the movement of photogenerated carriers, temperature-dependent phenomena are widely applied in both improving photodetection performance and reducing dark current. As depicted in Figure 5a, photocurrent density in InSb photodetector increases by two orders of magnitude when cooling from 300 K to 100 K [137]. Significant enhancements in detectivity and the suppression of noise current through cooling ZnCdSe/ZnCdMgS quantum well photodetectors have been observed in Figure 5b [35], increasing about five orders of magnitudes. The same phenomenon exists in the ZnCdSe/ZnCdMgS quantum cascade type [138]. Moreover, the cooling method can improve the light-electricity conversion efficiency, enabling the development of self-powered detectors. In Figure 5c, detectivity shows negative T-dependent and mobility-related behavior, owing to suppressed phonon scattering. A similar rule works on III-V group-based photodetectors. Wu et al. measured the quantum efficiency spectra of type-II InAs/InAsSb superlattice infrared photodetectors, which increased from 39% at 300 K to 47% at 150 K [126]. The temperature dependence of quantum efficiency mainly owes to the enhancement of the absorption coefficient and changes in resistance, while the recombination mechanism dominates the dark-current reduction. Moreover, these photoelectric performances show negative temperature dependence, indicating well-enhanced signals at temperatures lower than room temperature.

One of the unique advantages of low-temperature cooling in photovoltaic photodetectors is the capability of inhibiting dark current in diodes. In prevalent infrared photodetectors, the dark current is derived from majority carrier drift, G-R, defects-related leakage, carrier diffusion and the tunnelling process [141,142,143]. Depicted in Figure 5d, the relationship between resistance area product R_0_A, which represents dark current, and temperature experiences four stages in InAs/GaSb devices [139]. For these devices, the carrier diffusion process with activation energy becomes the main source of dark current from 300 to 160 K, showing the greatest temperature dependence. At the 100 to 140 K temperature range, the dominant mechanism turns to the G-R mechanism, which is dominated by Shockley–Read–Hall (SRH) processes in the depletion region, where dark current drops fast with declining temperature. From 25 to 120 K, small activation energy indicates that the defect-related leakage mainly contributes to dark current, which is less sensitive to temperature. If the temperature declines to near 10 K, the tunneling process dominates and decreases dark current further. Similar research on InGaAs/GaAsSb also found decreasing trap density with lowering temperature, accounting for the suppressed dark current [144]. Except for the III-V group photodetectors [145], the effectivity of temperature-controlling dark current has also been verified by studies on other photovoltaic photodetectors, such as HgTe CQDs [53], InSb/Si [137], β-FeSi_2_/Si [146], HgCdTe [101] and so on.

Moreover, the cooling process can simultaneously enhance performance through regulating absorption and carriers’ motion. For the temperature-dependent *J-V* characteristics, the open circuit voltage, listed in Table 1, in an ideal p-n junction can be addressed with a more specific formula [53]:

(6) $$\mathrm{Voc}=\frac{E_{g}}{e}-\frac{k_{b}T}{e}\ln\left(\frac{N_{c}N_{v}}{\mathrm{np}}\right)\;$$

where N_c_, N_v_ is the conduction band or valence band density of states and n/p is the electron/hole concentration. The negative relationship between V_OC_ and temperature is responsible for the increased efficiency at low temperature [21]. Jia et al. demonstrated the phenomenon of increasing photocurrent density with temperature decreasing in InSb photodetectors, ascribing it to longer carrier lifetime at lower temperatures [137]. A similar report on CdS/PbSe demonstrated longer carrier diffusion length through a cooling process [19]. Likewise, photovoltaic photocurrent is reported to increase with temperature decreasing, as shown, for example, in Figure 5e. The negative T-coefficient of photocurrent in MAPbI_3_ diodes is likely due to hole mobilities [140]. Based on the T-behavior of mobility, both acoustic phonon scattering and MA polarization disorder are responsible for the activation of mobility. Another key parameter of carrier motion is diffusion length or charge lifetime, shown in Figure 5f. McClintock et al. reported a rapid increase in diffusion length when temperatures cool to 80 K and assumed increased exciton fraction as an inducement [37]. Investigation of HgTe QDs found the same negative temperature coefficient of mobility due to cooling reducing tunneling barriers within QDs [147]. In general, the cooling enhanced photocurrent is based on the integration of carriers’ motion, carriers’ density and trap density.

Furthermore, cooling-enhanced photovoltaic devices with lateral photovoltaic effect (LPV) and bulk voltaic effect (BPV) are investigated. The position responsivity of ITO/Si has been researched by Qiao et al. showing 7.47 mV/mm at 80 K. This is about 1.91 times of performance at 295 K [148], attributed to different Schottky barriers at various temperatures. Previous reports on the BPV effect in PbZr_0.2_Ti_0.8_O_3_ (PZT) have observed increasing V_oc_ and decreasing dark current due to the temperature-influenced resistance of the Schottky barrier at ferroelectric-electrode interfaces [52].

#### 3.2.3. Photogating Photodetectors

**Figure 6:**
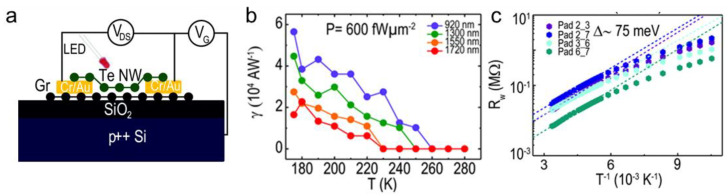
Cooling-enhanced phenomenon on photogating photodetectors. (**a**) The structure of photogating Te NW devices. (**b**) The temperature-dependent responsivity of Te NW devices. Reproduced with permission. (**c**) The temperature dependent-resistance of Te NW devices (adapted from [80], with permission from the Royal Society of Chemistry, 2017).

For 2D layered materials and their hybrid nanostructures, the photogating effect is a common mechanism that modulates structures’ photoconductivity through photogenerated local electric fields. Different from the linear relationship of photocurrent and light intensity in photoconductive mode, the photogating sensor performs with a sublinear tendency due to the underlying long-lived trap states [58]. Temperature-dependent research on photogating photodetectors is relatively lacking. Recently, Pradhan et al. fabricated a TeNW/graphene structure evidencing the feasibility of cryogenic-operation-enhanced optoelectronic performance [80], shown in Figure 6a. The photoresponsivity, represented as γ in Figure 6b, increases with temperature declining for the tested wavelengths, suggesting suppressed thermal fluctuation at low temperatures. To explain this phenomenon, authors also measured the variation of resistance with temperature in Figure 6c. Under the cooling condition, higher resistance is beneficial in reducing the leakage of photoinduced charges through source and drain electrodes. In consequence, cooling functions on the system’s resistance then improves photogating performance. In addition to regulations on the composition and morphology of 2D photogating semiconductors, the cooling method is promising regarding finely regulating photoresponse in photogating devices.

In conclusion, the cooling method is an effective way to enhance photodetection performance with impacts on absorption, photoconductivity, photogenerated carriers’ movement and more. Herein, we summarize the reported low-temperature-enhanced photoresponse in Table 2. According to the discussion above, 2D materials and infrared photodetectors possessing fewer defects have potential for low-temperature enhancement, suggesting further application in space and polar regions.

### 3.3. Performance of Solar Cells

**Figure 7:**
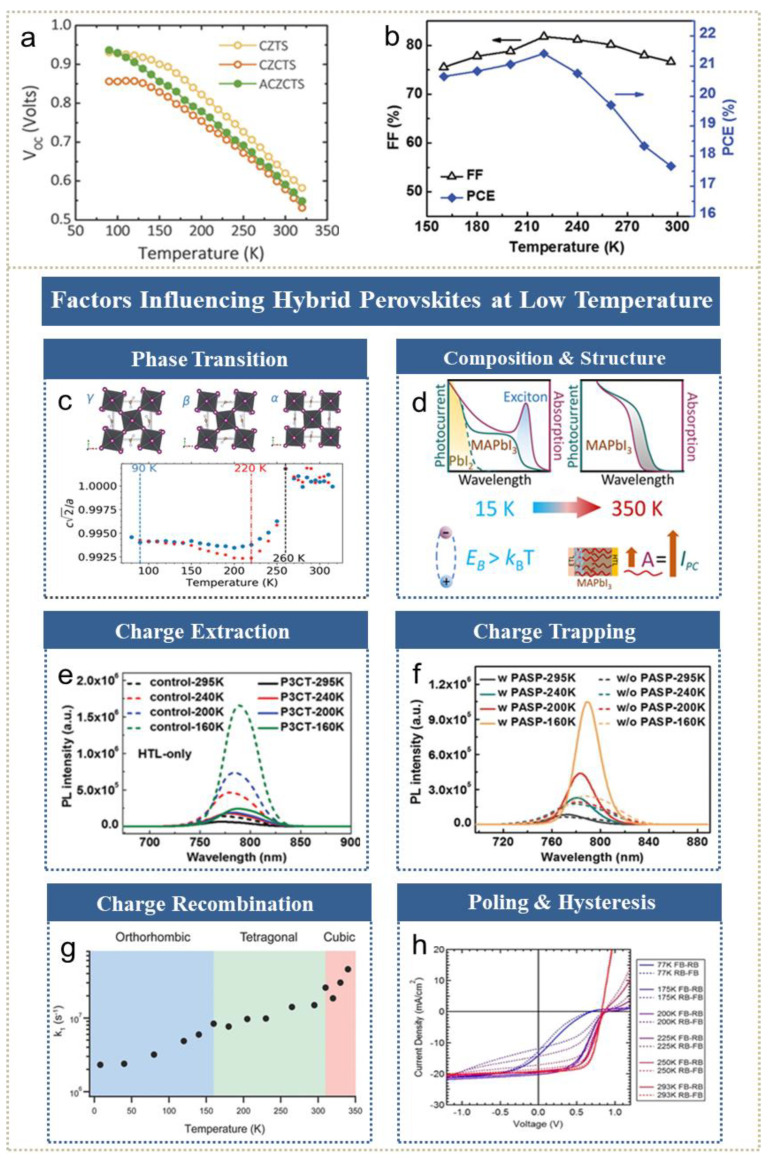
Low-temperature enhanced phenomena on solar cells. (**a**) Temperature-dependent V_OC_ of CZTS-based solar cells (adapted from [57], with permission from Wiley-VCH, 2018). (**b**) Cooling-enhanced PCE and FF of perovskite solar cells (adapted from [164], with permission from Wiley-VCH, 2019). (**c**) Phase transition (adapted from [39], with permission from Wiley-VCH, 2019). (**d**) Composition and structure (adapted from [165], with permission from Wiley-VCH, 2018). (**e**) Charge extraction. (**f**) Charge trapping (adapted from [164], with permission from Wiley-VCH, 2019). (**g**) Recombination rate (adapted from [30], with permission from Wiley-VCH, 2015). (**h**) Poling and hysteresis (adapted from [166], with permission from the American Chemical Society, 2015).

The capability of converting solar energy to applicable electronic energy renders great attention to solar cells, especially for low-cost solution-processed types [161]. Thin-film solar cells, such as perovskite and CZTS, yield remarkable power conversion efficiencies (PCEs) while still being behind the predicted Shockley–Queisser (S–Q) limit [162]. Moreover, stability in severe environments puts forward strict requirements for the balance between application and performance. Low-temperature operation is expected to reach its aims based on the intrinsic photoelectric mechanism. Figure 7a shows that V_OC_ varies with the temperature of CZTS-type solar cells [57], owing to higher minority carriers’ lifetime at low temperature. Taskensen et al. also witnessed the simultaneous improvement of V_OC_ and fill factor (FF) on CZTSe solar cells during cooling to 240 K [163]. As illustrated in Figure 7b, both the FF and PCE of hybrid perovskite solar cells significantly increase with declining temperature [164]. Temperature-dependent photovoltaic properties are closely related to light absorption, the motion of photogenerated carriers and even the structure and composition of devices.

Among thin-film solar cells, organic-inorganic hybrid perovskite solar cells have been reported to have advantages in a superior absorption coefficient, less excitons banding energy and an optimal energy level [166]. The cooling method has been utilized to explore the photoelectric process of perovskite materials and enhance their photoelectric output. To demonstrate the working mechanism of cooling, we summarize the most representative factors influencing hybrid perovskite solar cells’ performance at low temperature in Figure 7c–h. As mentioned before, both the PL maximum and absorption onset wavelength of organic-inorganic hybrid perovskite solar cells show a discontinuous behavior, suggesting the existence of phase transition. For the methyammonium halide perovskite type, as early as 1987, Poglitsch et al. observed the phase transition behaviour of MAPbCl_3_, MAPbBr_3_ and MAPbI_3_ from a cubic to an orthorhombic crystal structure [167], referred to as from α-phase to β-phase and finally γ-phase, through lattice data when temperature decreases. There are also investigations focusing on the dynamic theory of phase transition [168,169,170], not discussed in detail here. In response to in-depth knowledge of the interplay between low-temperature-caused structure transition and optoelectronic properties, Greenland et al. verified the dominated phase of mixed-cation mixed-halide perovskite (FAPbI_3_)_0.85_(MAPbBr_3_)_0.1_ at different temperatures and observed sudden changes in absorption [39] and carrier density, as well as the recombination rate at the corresponding phase-transition temperature, shown in Figure 7c. Lou et al. reported the temperature-dependent spectral response of FAPb(Br_0.4_I_0.6_)_3_. They also reported that β-phase, which possess fewer defects and a narrower band gap, appears at low temperature [171]. Moreover, accompanied by phase transition, an obvious change in the trend of temperature-dependent mobility has been observed at transition temperatures [172]. Landi et al. systematically studied the temperature dependence of recombination trap density and the fluctuating trap density of MAPbI_3_, showing less trap concentration at low temperatures and obvious turning points during the phase transition region [31]. Another study by Parrott et al. extracted the effective PL lifetime of FASn_x_Pb_1−x_I_3_, with x = 0.25 and 1, which increased with temperature reductions at each crystal-structure region, based on temperature-regulated recombination mechanisms [118]. Nevertheless, this experiment revealed that lead-rich (x < 0.5) perovskite possesses higher lifetime and increased stability compared with tin-rich (x > 0.5) perovskite, which is disturbed by natural defects. In addition to composition, pseudohalide additives are reported to enhance performance at low temperature by suppressing halide segregation [173]. Milot et al. further increased the effective charge-carrier mobility of FASnI_3_ at low temperature through the addition of SnF_2_, since hole-doping reduced recombination [36]. As Figure 7d illustrated, low-temperature enhancement testing of MAPbI_3_ showed remnant PbI_2_ contributing to higher photocurrent, especially at low temperatures [165]. Similar to enhancing methods at room temperature, charge-selective extraction plays an important role in enhancing cooling performance. Chen et al. studied cooling-caused variations in MAPbBr_3_ solar cells and proposed the insulating nature of a charge-extraction layer, rather than an absorption layer restricting performance at low temperature [174]. Represented by Figure 7e, the PL intensity of the charge-transporting layer is lowered compared to control samples at various temperatures, implying a more efficient charge transfer in interfaces, especially at low temperature [164]. Nevertheless, Shao et al. observed that deteriorated charge-carrier extraction caused a PCE drop at low temperature when using [60] PCBM as an electron extraction layer [103]. Subsequent experiments using a composite layer with higher electron extraction capability successfully raised PCE from 15.48% at 300 K to 17.72% at 215 K and 15.82% at 160 K. Furthermore, charge-extraction layers, like planar or mesoporous, shows different cryogenic-enhanced behaviours [175]. The fourth factor is trap states in perovskite solar cells. Piana et al. proposed that trap states influenced the trapping and re-excitation of free carriers and excitons and found the trap-states’ density maximum appearing at the tetragonal-orthorhombic phase transition temperature in MAPbI_3−x_Cl_x_ [38]. The rates of trapping declined from ~10^−8^ cm^3^s^−1^ at room temperature to ~10^−9^ cm^3^s^−1^ at low temperature due to less phonon-mediated recombination. Another experiment by Zhou et al. claimed the impact of surface passivation by PASP benefiting the reduction of trap states during temperature-dependent measurements [164]. The temperature dependence of charge-carrier recombination opens up a new mechanism for improving perovskite solar cells’ output. As mentioned in Table 1, monomolecular recombination (*k*_1_), biomolecular recombination (*k*_2_) and Auger recombination (*k*_3_) dominate the recombination mechanisms of perovskite solar cells. Depicted in Figure 7g, *k*_1_ decreased from 4.6 × 10^7^ s^−1^ at 340 K to 2.3 × 10^6^ s^−1^ at near 10 K in MAPbI_3_ devices. Since the low temperature passivates and freezes the dopant site, perovskite materials weakened by defects have promise of significant improvement at lower temperature. Different from *k*_1_, *k*_2_, which was evaluated with the Langevin model [176], generally exhibited a positive relationship with charge-carrier mobility, showing a low-temperature deteriorated trend. Recently, Davis et al. utilized the van Roosbroeck–Shockley relation to study the bimolecular recombination constant and found screening of the interactions between the electron-hole above the Mott transition to have potential in reducing *k*_2_, providing a feasible way to improve biomolecular recombination-restricted performance at low temperature [17]. Investigations on *k*_3_ reveals it is strongly dependent on carrier density [109] and monotonically increases as temperature declines [177]. The fluctuation of recombination composition brings pros and cons for cooling enhancement, which deserves in-depth research to reduce the overall recombination rate. Another breakthrough due to low-temperature operation is the hysteresis phenomenon. The hysteresis effect, originating from surface trap states, ferroelectricity and ion motion under applied bias [170,178,179,180], can be alleviated by freezing and bias poling. This is expected to promote the commercialization of perovskite solar cells. In temperature-dependent *J-V* scans, plotted in Figure 7h, Bryant et al. addressed the low-temperature-retarding relaxation process in hysteresis effects for 0.6 s at room temperature to 15.5 s at 175 K [166]. The influence of cooling on the hysteresis loop is based on competition between ions frozen by lowering temperature and the thermal-activated ion transporting, trapping and detrapping process [181]. Zou et al. combined bias poling and temperature-dependent hysteresis by applying positive bias and cooling to freeze the perovskite to a steady poled state [55], which increased the fill factor from ~0.3 at room temperature to ~0.7 at 160 K.

In general, low-temperature operation for solar cells is a rare but effective attempt. This method is connected with temperature-dependent changes in morphology, structure and overall carrier-charge motion. Development of cooling method can improve light-electricity conversion efficiency and application in extremely cool circumstances.

## 4. Applications of the Low-Temperature-Enhanced Photoelectric Process

**Figure 8:**
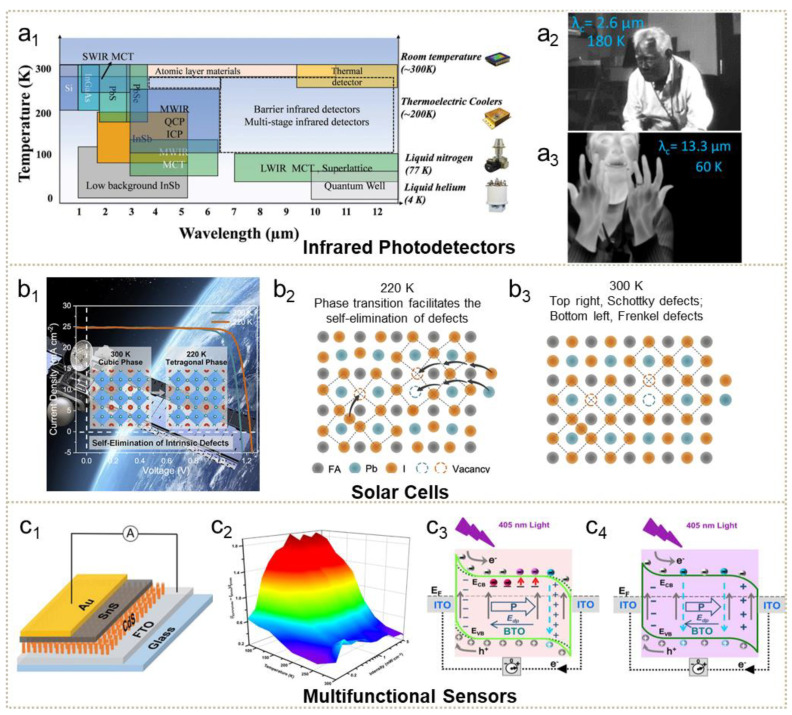
Applications of the low-temperature cooling method for photoelectric devices. (**a**) Cooling application in infrared photodetectors. (**a_1_**) Summary of working temperature for infrared photodetectors (adapted from [143], with permission from Wiley-VCH, 2019). (**a_2_**,**a_3_**) Temperature-dependent sensing performance of infrared photodetectors (adapted from [66], with permission from the Multidisciplinary Digital Publishing Institute, 2020). (**b**) Cooling application in solar cells (adapted from [181], with permission from Elsevier, 2020). (**c**) Cooling application in multifunction sensors. (**c_1_**,**c_2_**) Cooling-enhanced pyro-photoelectric coupling effect (adapted from [194], with permission from Wiley-VCH, 2020). (**c_3_**,**c_4_**) The underlying cooling-enhanced mechanism of the coupling effect (adapted from [197], with permission from Elsevier, 2019).

Advances in cooling-enhanced photoelectronic processes bring new opportunities to conventional photoelectrical nanomaterials, based on superiority in tuneable band alignment, suppressed noise current, a different mechanism of charge motion and so on, hence, enabling more innovative optoelectronic applications. The cooling method has been widely used in the photodetection field, especially for background-limited infrared photodetectors. As shown in Figure 8(a_1_), summarised by Wang et al., typical high-resolution photodetectors in the infrared field are dominated by materials with low-temperature operating requirements [143]. In addition to reduced dark current at cryogenic temperatures, significant enhanced detectivity makes the cooling method a more effective strategy for realizing highly sensitive photodetection. As shown, for example, by Figure 8(a_2_,a_3_), the III-V group photodetector acquires accuracy to distinguish details such as vessels when the temperature further drops to 60 K [66]. Despite difficulties in operation at extremely low temperature, the negative relationship between the temperature and the photoresponse of the discussed materials provides feasibility in greatly increasing devices’ performance using the proper cooling process. In contrast to the mature application of cooling in photodetection, solar cells possessing complex configuration and integrated optoelectrical mechanisms are in the beginning stage. Recently, Chen et al. successfully fabricated an unencapsulated perovskite device exhibiting higher performance in a simulated near-space (especially low-temperature) environment [181]. The enhancement at low temperature mainly originates from the self-elimination of intrinsic defects brought by phase transition under a cooling environment. As illustrated in Figure 8b, when temperatures decrease from 300 K to 220 K, the phase transition from cubic to tetragonal structure eliminates these intrinsic point defects then causes PCE to increase to 25.2% at 220 K. Previous discussion also lists the utilization of the frozen hysteresis effect at suitable temperature to improve the conversion efficiency of perovskite solar cells. Further investigations are expected to demonstrate the enormous advantages of cooling-enhanced solar cells, especially perovskite in space, near polar regions and other cool conditions. Integrated optoelectronic effects with other sensing functions play an important role in next-generation communication, healthcare, bioelectronics and so on [23,182,183,184,185,186,187,188,189,190,191,192,193]. In 2020, Chang et al. investigated the low-temperature cooling-enhanced pryo-phototronic effect on SnS/CdS photodetectors, depicted in Figure 8(c_1_,c_2_). For this planar structure photodetector, significant enhancement (~500%) of the ratio of pyroelectric to total signal was achieved as temperature declined to 77 K [194]. Further research on ZnO/perovskite [195] and p-Si/n-ZnO [196] verified that low temperature improved the coupling effect phenomenon. The coupling mechanism was unveiled by Ma et al. with experiments on BaTiO_3_ [197], shown in Figure 8(c_3_,c_4_). Under low-temperature operation, shallow trap levels play a more important role in photoinduced response. At room temperature, the function of shallow traps has been suppressed. However, the temperature dependence of piezo-photoelectric effect showed that cooling weakens piezoelectric and photoelectric performance [198,199]; these results remain a puzzle in the investigation of photoelectricity-evolved coupling mechanisms at low temperature. More multifunctional detectors operating at low temperature deserve investigation, especially those based on low-temperature-enhancing mechanisms. Another challenge for cooling operations is the limit of difficulties in realizing extremely low temperatures. According to specific temperature-dependent effects of optoelectronic devices, it is recommended to lock in optimal performance by cooling to the proper temperature rather than extreme low temperature. For the negative temperature-dependent section, even slightly cooler temperature will cause invisible improvement. In this regard, we believe that investigations on cooling-enhanced performance will make breakthroughs in low-temperature photoelectric application and push forward research on temperature-regulated optoelectrical mechanisms.

## 5. Summary and Outlook

In this review, we propose a low-temperature operation method with huge potential in photodetectors and solar cells without altering nanostructures or configurations and summarize the relevant advances of cooling methods in improving optoelectrical performance. Advances on cooling-enhanced photoelectric performance have proven its benefits to various materials and structures, for example, 2D photoconductors, III-Vs group infrared photodetectors and perovskite solar cells. In addition to the summary of cooling-enhanced photoelectric performance, in-depth mechanisms have been discussed in terms of different application types, represented by photodetectors and solar cells. Through analysis from materials to performance, the cooling method is based on the integration of bandgap optimization, recombination suppression, phase transition and so on. Moreover, there is also plenty of room to further improve photoelectric performance and realize practical applications of properly lowering temperature. Overall, the low-temperature cooling method, which lacked attention in recent decades, is capable of pushing forward photoelectric applications in harsh environments and featuring highly improved optoelectronic performance without complex modification.

## Figures and Tables

**Table 1:** Photoelectric parameters with certain temperature-dependent relationships.

Parameter	Symbol	Unit	T-Dependent	Ref
Bandgap	E_g_	eV	$E_{g}=E_{0}-\frac{{\alpha T}^{2}}{T+\beta }$	[105]
Diffusion length	L_D_	μm	$L_{D\left(n\right)}=\sqrt{\frac{{\mu k}_{B}T}{{\mathrm{eR}}_{\mathrm{total}}\left(n\right)}}$	[30]
Open circuit voltage	V_oc_	V	$V_{\mathrm{OC}}=\frac{\mathrm{nkT}}{e}\ln\left(\frac{J_{\mathrm{PH}}}{J_{o}}\right)$	[93]
Reverse saturation current	J_0_	mA cm^−2^	Thermionic emission model: $J_{\mathrm{TE}}={\mathrm{AT}}^{2}\exp\left(-\frac{q\phi_{B}}{\mathrm{kT}}\right)\left[\exp\left(\frac{\mathrm{qV}}{\mathrm{kT}}-1\right)\right]$	[106]
			Diffusion model: $J_{\mathrm{Diff}}={e\mu }_{e\left(h\right)}N_{C\left(V\right)}E_{m}\exp\left(\frac{-e\phi_{B}}{\mathrm{kT}}\right)$	
Photoconductivity	σ	S cm^−1^	Variable Range Hopping: $\sigma_{1}=\sigma_{0}\exp\left[-\left(\frac{T_{0}}{T}\right)\frac{1}{4}\right]$	[107,108]
			Nearest Neighbor Hopping: $\sigma_{2}=\left[\frac{v_{0}e^{2}c\left(1-c\right)}{\mathrm{kTr}}\right]\exp\left(-2\alpha r\right)\exp\left(\frac{-\Delta E}{\mathrm{kT}}\right)$	
Recombination rate	R_total_	/	$R_{\mathrm{total}}=-k_{\mathrm{Auger}}n^{3}-k_{\mathrm{bimo}}n^{2}-k_{\mathrm{mono}}n$	[109]

**Table 2:** Summary of cooling-enhanced photodetection performance.

Materials	Type	Wavelength	300 K		Cryogenic		Ref
			R (A/W)	D (Jones)	R (A/W)	D (Jones)	
graphene–TeNW	PG	920–1720 nm	5	5 × 10^13^	5.7 × 10^4^		[80]
Te	PC	1–3.4 μm	16	2 × 10^9^	27	2.6 × 10^11^	[47]
Black-Phosphorus	PC	400–900 nm	~10^3^		7 × 10^6^		[78]
A-GNRs ^(a)^				~2.2 × 10^8^		~2.1 × 10^11^	[81]
HgTe QDs	PC	3–5.25 μm	~0.001	~10^7^	~0.1	~5 × 10^10^	[149]
HgTe/Ag_2_Te CQDs	PV	4–5 μm	~0.1	~3 × 10^8^	0.56	~10^11^	[53]
HgTe CQDs	PV	3–5 μm		7.2 × 10^8^	1.46	4 × 10^11^	[102]
PbSe CQDs	PC			~1 × 10^13^		8.1 × 10^13^	[150]
Sb_2_Te_3_	PC	~980 nm	~0.5		~3.5		[128]
Te-hyperdoped Si	PV	3–5 μm		~10^9^		~10^11^	[151]
InAs/InAs_1-x_Sb_x_	PV	~4.75 μm	0.78	~10^9^	~0.7	2.9 × 10^12^	[152]
InAs/GaAs	PV	2.5–7 μm	~3	2.4 × 10^8^	~27	2 × 10^9^	[153]
InAs/Si	PC	1.4–3 μm		1.4 × 10^5^	60.4 × 10^−3^	6 × 10^7^	[154]
InAs/GaSb	PV	~7 μm	~0.14	8.9 × 10^8^	0.167	3.0 × 10^11^	[155]
InAs/InP	PV	3–5 μm		6 × 10^7^	0.822	2.8 × 10^11^	[156]
InAs/InAsSb	PV	~5.1 μm		2.5 × 10^9^		7.1 × 10^11^	[126]
InAs/AlAsSb	PV	4–5 μm	1.9 × 10^−3^	2.7 × 10^7^	~6.8 × 10^−3^	~10^9^	[63]
InAsSb/InSb	PV	~4.6 μm		~5 × 10^9^		6 × 10^11^	[157]
InAs/GaAs/InGaAs/InAlAs	PV	3–5 μm	0.6 × 10^−3^	4.83 × 10^6^	1.21 × 10^−3^	3.64 × 10^11^	[158]
Ge/Ge_0.975_Sn_0.025_/Ge	PV	~2 μm	~0.1		~0.3		[14]
Ge_0.9_Sn_0.1_/Si	PV	~2.4 μm	0.26		2.85	4 × 10^9^	[159]
Bi_2_O_2_Se	PC	UV–NIR	6.5	8.3 × 10^11^	1.7	~1.3 × 10^12^	[160]
Ta_2_NiSe_5_	PC		17.21		62.22		[129]
Pb_1−x_Sn_x_Se	PC	3–5 μm	0.21	1.1 × 10^12^	0.19	7.7 × 10^13^	[51]
ZnCdSe/ZnCdMgSe	PV	3–8 μm	~0.2 × 10^−3^	10^5^	40 × 10^−3^	3.1 × 10^10^	[138]
Zn_0.51_Cd_0.4_Se/Zn_0.29_Cd_0.26_Mg_0.45_Se	PV	3–5 μm	30	4 × 10^7^		2 × 10^9^	[35]
Fe_3_(THT)_2_(NH_4_)_3_	PC	400–1575 nm		3 × 10^8^		7 × 10^8^	[20]

^(a)^ A-GNRs: Armchair graphene nanoribbons.

## Data Availability

No new data were created or analyzed in this study. Data sharing is not applicable to this article.
